# Diet–gut microbiota–immune–brain interactions in aging: mechanistic pathways and clinical implications

**DOI:** 10.3389/fnmol.2026.1847644

**Published:** 2026-08-18

**Authors:** Maha A. Jarfan, Karani Santhanakrishnan Vimaleswaran, Anisha Wijeyesekera

**Affiliations:** Department of Food and Nutritional Sciences, University of Reading, Reading, United Kingdom

**Keywords:** cognitive function, diet, gut microbiota, gut–brain axis, immune signaling, neuroinflammation, personalized nutrition, short-chain fatty acids

## Abstract

With rising life expectancy and global population aging, cognitive decline has become a major and growing public health challenge. Advances in nutritional neuroscience highlight the gut microbiota–immune–brain axis as a key biological pathway through which diet may influence cognitive function during aging. The gut microbiota, a metabolically active ecosystem, responds dynamically to habitual dietary patterns and produces bioactive metabolites capable of modulating immune signaling, neuroinflammation, and neuronal function. Diets rich in microbiota-modulating foods (e.g., dietary fiber, polyphenols, prebiotics, and probiotics) promote beneficial microbial communities. These communities support short-chain fatty acid production, maintain intestinal barrier integrity, and regulate systemic immune responses, processes increasingly associated with cognitive resilience in aging populations. In contrast, Western-style dietary patterns characterized by high intakes of saturated fats and refined sugars are linked to microbial dysbiosis, impaired gut barrier function, metabolic endotoxemia, and chronic low-grade inflammation, which may contribute to neuroinflammatory pathways involved in cognitive decline. This narrative review synthesizes evidence from observational studies, dietary intervention trials, and mechanistic animal models to examine how diet-driven alterations in gut microbiota composition and microbial metabolites interact with and modulate immune pathways to influence brain function in aging populations. Although emerging evidence supports the biological plausibility of this axis in cognitive health, current evidence remains constrained by methodological heterogeneity, short intervention durations, limited functional insight, and substantial inter-individual variability in microbiota responsiveness. In particular, much of the mechanistic understanding derives from preclinical research, while human evidence remains largely associative and insufficient to establish causal pathways. Future research should integrate longitudinal cohort designs, harmonized cognitive assessment tools, and repeated profiling of microbial and host metabolites to clarify the functional and causal links between diet, microbial metabolism, immune regulation, and brain aging. A more integrated understanding of these interactions may help inform targeted, microbiome-informed nutritional strategies for supporting healthy cognitive aging, while maintaining appropriate caution in clinical interpretation.

## 1 Introduction

The human gut microbiota is increasingly recognized as a central regulator of host physiology, influencing metabolic, immune, and neurobiological processes that extend beyond the gastrointestinal tract. Through its metabolic capacity and continuous interaction with host signaling pathways, the gut microbiota contributes to systemic homeostasis and plays a key role in shaping brain function across the lifespan. Increasing evidence suggests that microbial metabolites, immune mediators, and neuroendocrine signals derived from the gut influence central nervous system function, positioning the gut microbiota as a key component of the microbiota–gut–brain axis (Cryan et al., 2019).

At the same time, global population aging is accelerating at an unprecedented rate. Age-related cognitive decline and dementia represent major and growing public health challenges worldwide. Recent estimates indicate that over 55 million people were living with dementia globally in 2020, with the number projected to reach approximately 139 million by 2050 as populations continue to age (Alzheimer’s Disease International, 2020; Nichols et al., 2022; World Health Organization, 2021). These trends underscore the urgency of identifying modifiable lifestyle factors that support cognitive health and delay neurodegenerative processes. Among these factors, diet has emerged as a particularly important determinant, given its capacity to influence both brain physiology and the composition and metabolic activity of the gut microbiota (Livingston et al., 2020; Scarmeas et al., 2006).

The gut–brain axis represents a complex bidirectional communication network linking the gastrointestinal tract with the central nervous system through neural, endocrine, immune, and metabolic signaling pathways (Cryan et al., 2019). Within this system, the gut microbiota functions as a metabolically active ecosystem that participates in nutrient metabolism, immune regulation, and maintenance of intestinal barrier integrity. One of the most important metabolic activities of the gut microbiota is the fermentation of dietary fibers into short-chain fatty acids (SCFAs), primarily acetate, propionate, and butyrate. These microbial metabolites contribute to intestinal barrier integrity, regulate immune signaling, and influence neurophysiological processes that may affect brain function (Koh et al., 2016).

Emerging evidence suggests that microbiota-derived metabolites can interact with host immune pathways and influence systemic inflammatory responses. Chronic low-grade inflammation, often described as “inflammaging,” has been increasingly recognized as a key biological process contributing to age-related cognitive decline and neurodegeneration (Franceschi et al., 2018). In this context, alterations in gut microbial composition, commonly referred to as dysbiosis, have been associated with metabolic disorders, immune dysregulation, and neurodegenerative diseases, including Alzheimer’s disease and Parkinson’s disease (Fan and Pedersen, 2021; Marchesi et al., 2016).

Dietary patterns represent one of the most powerful environmental determinants shaping the composition and metabolic output of the gut microbiota (Sonnenburg and Bäckhed, 2016). Diets rich in fiber, fermented foods, prebiotics, and plant-derived polyphenols promote microbial diversity and beneficial metabolic activity. In contrast, Western-style dietary patterns characterized by high consumption of saturated fats, refined sugars, and ultra-processed foods are associated with reduced microbial diversity, increased intestinal permeability, metabolic endotoxemia, and heightened inflammatory signaling (Christ et al., 2018; Makki et al., 2018; Sanders et al., 2019). These diet-induced microbial and immune alterations may represent an important mechanistic pathway linking nutritional exposures to cognitive health outcomes.

Despite rapid progress in gut microbiota research, several important knowledge gaps remain. Much of the current mechanistic understanding of microbiota–brain interactions has been derived from experimental animal models, which may not fully capture the complexity of human physiology, long-term dietary exposures, and environmental influences (Mayer et al., 2015). In addition, human studies investigating diet, gut microbiota, and cognitive outcomes often differ substantially in methodological approaches, microbiome profiling techniques, dietary assessment tools, and cognitive testing protocols, limiting the comparability and integration of findings across populations (Sinha et al., 2015). Addressing these limitations requires integrative frameworks capable of linking dietary exposures, microbial metabolism, immune signaling, and brain function within the broader context of host–microbiome interactions and cognitive aging.

**Figure 1:**
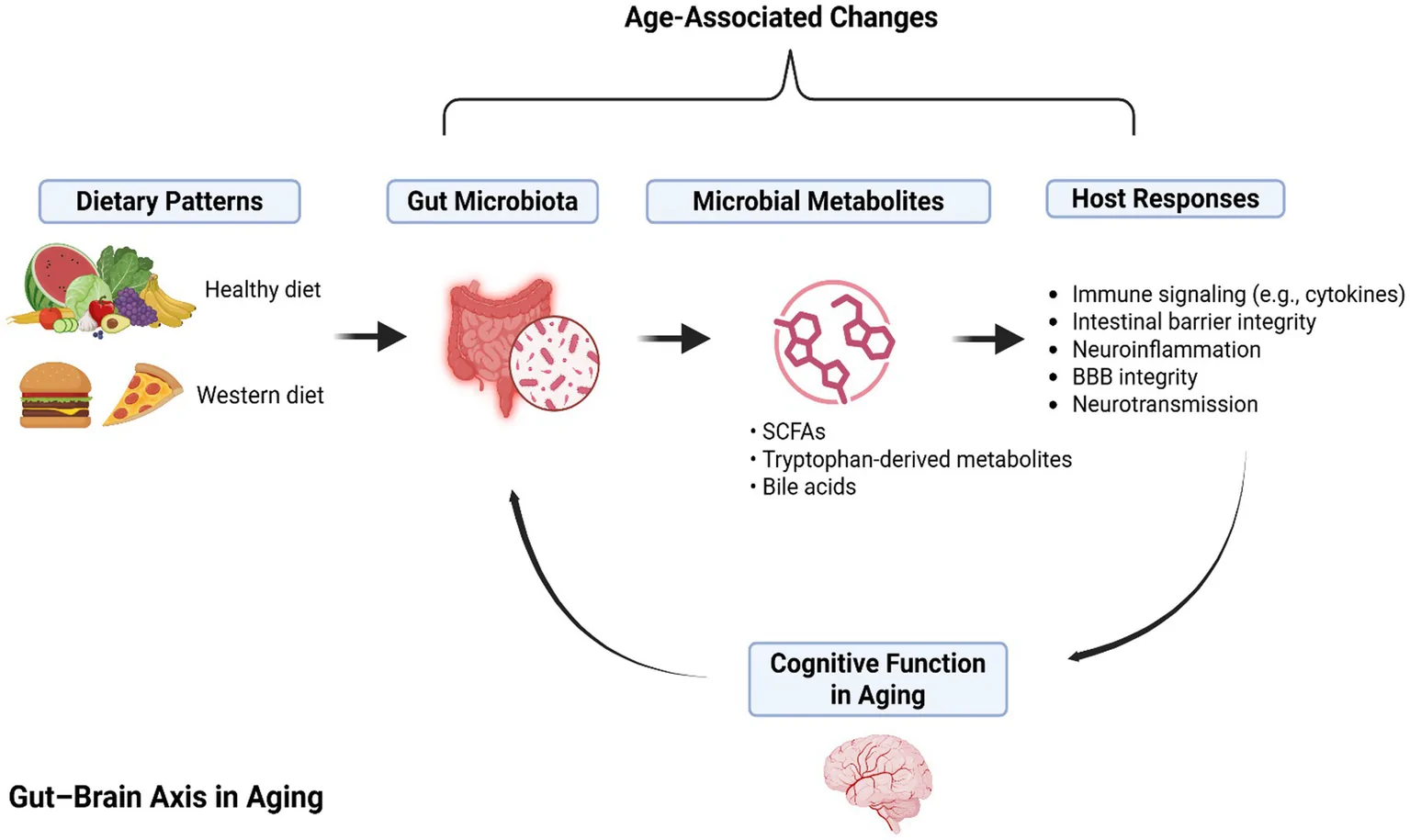
Conceptual overview of the diet–gut microbiota–immune–brain axis in aging. Dietary patterns shape the composition and metabolic activity of the gut microbiota, leading to the production of bioactive metabolites, including short-chain fatty acids (SCFAs), tryptophan-derived metabolites, and bile acids. Aging-associated changes influence gut microbiota composition, microbial metabolism, and host responses. These microbiota-derived metabolites interact with host pathways, modulating immune signaling, intestinal barrier integrity, neuroinflammation, blood–brain barrier (BBB) integrity, and neurotransmission. Through bidirectional communication along the gut–brain axis, these processes collectively influence cognitive function during aging. Created in BioRender. Jarfan, M. (2026). https://BioRender.com/a4nf96l.

Although the microbiota–gut–brain axis has been extensively reviewed, many existing reviews focus on individual signaling pathways, specific metabolite classes, or particular neurological disorders. Comparatively less attention has been given to how aging reshapes diet–microbiota–immune–brain interactions and influences the mechanisms through which dietary and microbial signals affect neuroinflammation and cognitive function. This review adopts an aging-focused perspective by integrating evidence from dietary, microbiome, immunological, and neurobiological research within a unified conceptual framework. Particular emphasis is placed on how microbiota-derived metabolites, including SCFAs, tryptophan-derived metabolites, and bile acids, interact with immune and neurobiological pathways relevant to cognitive aging. Importantly, rather than simply cataloging associations, this review critically evaluates the relative strength and limitations of mechanistic, observational, and interventional evidence across these interconnected pathways. By synthesizing these converging lines of evidence, the review aims to provide a framework for understanding how microbiota-informed nutritional strategies may support cognitive health and healthy aging. To facilitate conceptual clarity and integrate the multidimensional pathways described above, a schematic representation of diet–gut microbiota–immune–brain interactions in aging is presented in Figure 1.

## 2 Aging-associated gut microbiota remodeling and its consequences for gut–brain axis function

### 2.1 Age-related changes in gut microbiota composition

Aging is increasingly recognized as a biological process that actively reshapes the structure and function of the gut microbiota rather than merely providing the context for age-related diseases. Physiological changes, immune remodeling, lifestyle factors, dietary habits, and medication use collectively contribute to the remodeling of the intestinal microbial ecosystem, which is characterized by altered microbial diversity, shifts in commensal and opportunistic taxa, and changes in microbial metabolic activity. These alterations influence immune regulation, gut barrier integrity, and gut–brain communication, making age-related microbiome remodeling an integral component of the microbiota–gut–brain axis and a potential contributor to the heterogeneity of cognitive and neurological aging trajectories (Bradley and Haran, 2024; Cryan et al., 2019; DeJong et al., 2020; Ghosh et al., 2022).

One of the hallmark features of age-associated microbiome remodeling is altered microbial diversity and community structure. Although aging has traditionally been associated with reduced microbial diversity and increased inter-individual variability, recent evidence indicates that these changes are influenced more by health status than by chronological age alone. Reduced diversity is most consistently observed in frail, institutionalized, and medically compromised older adults, whereas healthy aging is associated with the maintenance of microbial diversity and increasingly individualized microbial communities. Supporting this concept, Wilmanski et al. demonstrated that healthy aging was associated with progressive microbiome uniqueness, a pattern that was absent in less healthy older individuals (Claesson et al., 2012; Wilmanski et al., 2021).

Aging is also associated with characteristic shifts in the abundance of specific microbial taxa. Declines in beneficial commensal microorganisms, particularly the butyrate-producing genera *Faecalibacterium* and *Roseburia*, together with *Bifidobacterium*, are among the most consistently reported findings. Conversely, frailty, multimorbidity, and institutionalization are associated with an increased abundance of opportunistic and potentially pro-inflammatory microorganisms, including members of the *Enterobacteriaceae* family and other pathobionts. These microbial signatures are not universal across all older adults; healthy aging is generally associated with the preservation of beneficial microbial communities and greater ecological stability, whereas frailty is linked to dysbiotic microbial profiles. Collectively, these observations emphasize that biological and functional health plays a greater role than chronological age in shaping age-related microbiome trajectories (Bradley and Haran, 2024; Ghosh et al., 2022; Jackson et al., 2016; Kossowska et al., 2024; Wen et al., 2025).

Collectively, current evidence indicates that aging is associated with coordinated alterations in microbial diversity, community composition, and ecological organization that influence the functional capacity of the gut microbiota. These compositional changes are accompanied by corresponding changes in microbial metabolite production, highlighting the importance of considering both taxonomic and functional characteristics when evaluating microbiome aging. Among the most important functional consequences is a reduced capacity for butyrate biosynthesis, together with alterations in the production of other health-promoting microbial metabolites, as discussed in the following section (DeJong et al., 2020; Ghosh et al., 2022).

### 2.2 Decline in microbial metabolic capacity during aging

Beyond alterations in microbial composition, aging is increasingly associated with a decline in the metabolic capacity of the gut microbiota. While taxonomic changes provide important insights into microbiome remodeling, the biological consequences of aging are primarily mediated through functional alterations in microbial metabolism. Emerging evidence indicates that aging is accompanied by reduced metabolic flexibility and a diminished capacity of microbial communities to produce bioactive metabolites that support host physiology. Consequently, age-related microbiome remodeling may disrupt host–microbe metabolic cross-talk, contributing to impaired immune regulation, intestinal homeostasis, and gut–brain communication. These functional alterations are increasingly recognized as important determinants of healthy versus unhealthy aging and help explain why microbial composition alone does not fully capture the biological consequences of microbiome aging (Ghosh et al., 2022; Rampelli et al., 2013).

One of the best-characterized functional consequences of microbiome aging is reduced production of short-chain fatty acids (SCFAs), particularly butyrate. This decline is partly attributable to age-associated reductions in key butyrate-producing genera, including *Faecalibacterium*, *Roseburia*, and *Eubacterium* (Ghosh et al., 2022; Jackson et al., 2016). In addition to taxonomic changes, aging is associated with remodeling of metabolic pathways involved in butyrate production. Early metagenomic studies reported reduced SCFA-related functional potential in extreme aging, whereas more recent shotgun metagenomic analyses suggest that aging is characterized by shifts in butyrate biosynthesis routes rather than a uniform loss of butyrate-producing capacity (Rampelli et al., 2013; Ravikrishnan et al., 2024). Consequently, older adults may exhibit an altered microbial capacity to generate butyrate and other SCFAs that are essential for maintaining intestinal homeostasis, epithelial barrier integrity, immune regulation, host metabolism, and gut–brain communication (Ghosh et al., 2022; Rampelli et al., 2013).

Declining microbial metabolic capacity is not solely attributable to the loss of individual taxa but also reflects disruption of the ecological interactions that sustain microbiome function. In healthy microbial ecosystems, cross-feeding networks enable metabolites produced by one microbial group to serve as substrates for others, a process that is particularly important for butyrate production through sequential metabolism by multiple bacterial taxa (Culp and Goodman, 2023). Aging has been associated with depletion of a co-abundant guild of health-associated microorganisms occupying central positions within microbial interaction networks, accompanied by enrichment of disease-associated taxa (Ghosh et al., 2022). In parallel, reduced functional redundancy has been proposed as a contributor to declining microbiome stability and metabolic performance, although direct evidence in aging populations remains limited (Tian et al., 2020). Together, these alterations may disproportionately impair community-wide metabolic output and host–microbiome metabolic cross-talk, increasing susceptibility to inflammation, metabolic dysfunction, impaired gut–brain communication, and age-related physiological decline (Culp and Goodman, 2023; Tian et al., 2020).

The consequences of declining microbial metabolic activity extend beyond the microbiota to directly influence host physiology. Reduced production of butyrate and other beneficial microbial metabolites may compromise intestinal epithelial barrier integrity, impair immune homeostasis, and alter immune, endocrine, and metabolic signaling pathways involved in host responses to microbial metabolites. These changes increase susceptibility to chronic low-grade inflammation and reduce physiological resilience during aging. Moreover, preclinical evidence suggests that impaired microbial metabolic activity disrupts gut–brain communication through downstream effects on immune, endocrine, and metabolic signaling, thereby contributing to neurobiological changes associated with aging (Mishra et al., 2024). Collectively, these mechanisms provide an important biological link between age-related microbiome remodeling and inflammaging, forming the mechanistic basis for the immune and neurobiological consequences discussed in the following section (Ghosh et al., 2022; Jackson et al., 2016).

### 2.3 Inflammaging and age-related gut barrier dysfunction

Inflammaging, the chronic low-grade systemic inflammation that develops during aging, is increasingly recognized as a hallmark of biological aging and has been implicated in the development of numerous age-related disorders, including cardiovascular disease, metabolic dysfunction, and frailty. Although inflammaging arises through multiple interacting mechanisms, accumulating evidence suggests that age-associated alterations in the gut microbiota contribute to this process. Changes in microbial composition, reduced production of beneficial microbial metabolites, and impaired host–microbiome interactions have all been proposed to promote immune dysregulation and persistent inflammatory activity. Consequently, the aging gut microbiome is increasingly viewed as an active contributor to the development and maintenance of systemic inflammation during later life (DeJong et al., 2020; Franceschi et al., 2018).

Age-associated alterations in microbial metabolic activity also have important consequences for intestinal barrier function. The integrity of the intestinal epithelium depends on coordinated interactions among epithelial cells, the mucus layer, immune components, and microbial metabolites, particularly butyrate. As a primary energy source for colonocytes, butyrate promotes mucus production through upregulation of *MUC* gene expression (Gaudier et al., 2004) and enhance tight junction assembly and epithelial barrier integrity (Peng et al., 2009). Age-related reductions in butyrate-producing microorganisms have been documented in older adults and are accompanied by broader alterations in microbial metabolic activity (Biagi et al., 2010; Claesson et al., 2012). Although experimental studies consistently support a barrier-protective role for butyrate, direct evidence demonstrating reduced butyrate availability as a primary driver of age-related intestinal permeability in humans remains limited (Tabat et al., 2020). Nevertheless, increased intestinal permeability has been reported in older adults and may facilitate the translocation of microbial products into the systemic circulation, thereby promoting chronic immune activation and inflammatory signaling (Buford et al., 2018; Qi et al., 2017; Stehle et al., 2012). Together, these findings provide a mechanistic link between age-related microbiome remodeling and inflammaging.

Compromised intestinal barrier function may increase exposure of the host immune system to microbial products that are normally confined to the intestinal lumen. Among these, lipopolysaccharide (LPS) and other microbial-associated molecular patterns have received particular attention. Experimental studies indicate that age-associated microbial dysbiosis may promote intestinal permeability and facilitate microbial translocation, thereby contributing to systemic inflammatory responses (Thevaranjan et al., 2017). In addition, the aged gut microbiota exhibits enhanced immunogenic properties, including increased activation of Toll-like receptor 4 (TLR4)-mediated signaling pathways (Caetano-Silva et al., 2024). Activation of TLR4 stimulates downstream inflammatory cascades and promotes the production of pro-inflammatory cytokines, including interleukin-6 (IL-6) and tumor necrosis factor-*α* (TNF-α). Persistent activation of these pathways is thought to contribute to the chronic immune activation characteristic of inflammaging (Franceschi et al., 2000). Consistent with this concept, elevated circulating IL-6 has been associated with subsequent disability and functional decline in older adults (Ferrucci et al., 1999). Human studies further support the role of intestinal barrier dysfunction by demonstrating increased circulating zonulin concentrations together with positive associations with TNF-α and IL-6 in healthy older adults (Qi et al., 2017). Although much of the mechanistic evidence derives from experimental models, collectively, current evidence suggests that age-related microbiome remodeling may promote intestinal permeability, enhance innate immune activation, and contribute to the chronic systemic inflammation that accompanies aging.

### 2.4 Aging and altered gut–brain communication

The gut–brain axis is a complex bidirectional communication network that integrates neural, endocrine, immune, and metabolic pathways linking the gastrointestinal tract and the central nervous system (Cryan et al., 2019). Beyond immune-mediated mechanisms, the gut microbiota communicates with the brain through direct neural pathways, with the vagus nerve serving as the principal afferent conduit linking intestinal signals to central neural circuits involved in autonomic regulation, emotional processing, and cognition. A major advance in understanding the gut–brain axis was the discovery that specialized enteroendocrine cells establish direct synaptic connections with vagal afferent neurons, enabling rapid transmission of gut-derived signals to the brain through neural rather than exclusively endocrine pathways (Kaelberer et al., 2018). More recent evidence indicates that microbiota-derived metabolites modulate vagal signaling primarily through receptor-mediated interactions with enteroendocrine cells. In addition, select metabolites may directly influence vagal sensory neurons in experimental mouse models (Jameson et al., 2025). Together, these findings demonstrate that neural gut–brain communication provides a mechanism through which microbial activity is continuously translated into signals reaching the central nervous system.

Aging may alter multiple components of this neural communication network. In humans, aging is associated with structural changes within the enteric nervous system, including progressive reductions in myenteric neuronal density, with evidence of relative preservation of nitrergic neuronal populations rather than selective targeting of specific neuronal subtypes (Bernard et al., 2009). Functional studies in aged mice have similarly demonstrated alterations in gut-to-brain neural signaling, including changes in the firing characteristics of enteric sensory neurons and their downstream vagal pathways (McVey Neufeld et al., 2024). These neural alterations occur alongside age-related gut microbiome remodeling and corresponding shifts in circulating microbially derived metabolites, including compounds involved in amino acid metabolism and host–microbiome signaling (Wilmanski et al., 2021). Collectively, these observations suggest that aging may influence both the structural integrity of the enteric nervous system and the molecular signals that support communication between the gut and the brain.

Recent mechanistic studies provide further evidence that age-associated microbiome remodeling can directly influence neural gut–brain communication. Experimental work in aged mice has shown that aging-associated microbial dysbiosis may impair vagal sensory signaling through microbiota-derived metabolites and immune mediators, resulting in altered hippocampal activity and memory impairment (Cox et al., 2026). Importantly, interventions targeting this pathway restored vagal signaling and improved cognitive performance in experimental models, suggesting that neural gut–brain communication may represent a modifiable component of age-related cognitive decline. However, the mechanistic evidence discussed above derives predominantly from murine models, and the extent to which these findings translate to human aging remains to be fully established.

Taken together, current evidence suggests that aging produces a coordinated remodeling of neural gut–brain communication, encompassing alterations in enteric nervous system structure, vagal signaling, and microbiota-derived neural modulators. These changes may alter gut-to-brain communication and increase the vulnerability of the aging brain to microbiota-driven disturbances, providing a mechanistic framework for understanding age-dependent susceptibility along the gut–brain axis.

### 2.5 Age-dependent vulnerability of the gut–brain axis in neurodegenerative disease

The most direct experimental evidence for this concept comes from studies of *α*-synuclein, the misfolded protein that characterizes synucleinopathies, including Parkinson’s disease. A landmark study by Challis et al. demonstrated that peripheral inoculation of α-synuclein preformed fibrils induced enteric nervous system pathology and gastrointestinal dysfunction in both young and aged mice, whereas propagation of α-synuclein pathology to the brainstem, accompanied by persistent motor deficits and striatal dopamine depletion, occurred selectively in aged animals (Challis et al., 2020). These findings indicate that the aged host environment provides a more permissive biological context for gut-to-brain propagation of pathological α-synuclein than the young host. This age-dependent vulnerability was subsequently confirmed in wild-type rats, where gastrointestinal administration of aggregated α-synuclein resulted in more extensive transneuronal spread and the formation of denser, more protease-resistant aggregates in aged animals, suggesting that aging influences both the efficiency of pathological transmission and the properties of the resulting protein aggregates (Van Den Berge et al., 2021).

What renders the aged gut–brain axis permissive to pathological transmission remains an active area of investigation, but several converging mechanisms have been identified. Earlier studies demonstrated that the gut microbiota is required for motor deficits, microglial activation, and α-synuclein pathology in α-synuclein-overexpressing mice, while colonization with microbiota from patients with Parkinson’s disease exacerbated motor impairment compared with microbiota from healthy donors, demonstrating a causal role for the gut microbiome in disease progression in an experimental mouse model (Sampson et al., 2016). Human metagenomic analyses subsequently confirmed widespread alterations in the Parkinson’s disease microbiome, characterized by enrichment of immunogenic and potentially neurotoxic taxa together with depletion of anti-inflammatory and neuroprotective taxa (Wallen et al., 2022). Collectively, these findings suggest that disease-associated microbial communities may contribute to neuroinflammation and increase susceptibility to neurodegeneration during aging.

A parallel mechanism through which the aging microbiome may increase susceptibility to neurodegeneration involves microglia, the brain’s resident innate immune cells. Comparative transcriptomic analyses demonstrated that the gut microbiota drives age-associated changes in microglial gene expression and that depletion of the gut microbiota ameliorates oxidative stress and mitochondrial dysfunction in aged microglia (Mossad et al., 2022). Metabolomic analyses identified N6-carboxymethyllysine (CML), a gut microbiota-dependent metabolite whose intestinal translocation is facilitated by age-related barrier dysfunction, as a mediator of microglial impairment. Elevated CML levels were subsequently validated in human serum and brain tissue during aging (Mossad et al., 2022). Together, these findings suggest that age-associated microbiome remodeling may impair microglial function through gut-derived metabolites that accumulate with age.

In Alzheimer’s disease, the evidence follows a similar pattern, although the mechanistic case for age-dependent gut–brain transmission is less direct. Fecal microbiota transplantation from healthy wild-type donors into transgenic mice with established amyloid and tau pathology reduced plaque burden, glial reactivity, cognitive impairment, and peripheral inflammation, demonstrating that the gut microbiome can causally modulate neuropathology through gut–immune–brain pathways (Kim et al., 2020). Human studies further indicate that gut microbiome alterations precede clinical disease, with cognitively normal individuals showing distinct microbial profiles associated with preclinical amyloid and tau pathology independent of dietary variation (Ferreiro et al., 2023). These findings suggest that gut microbiome remodeling may occur early in the Alzheimer’s disease trajectory, although whether it represents a causal driver or an early consequence of pathology remains to be determined by longitudinal studies.

The modifiability of these processes is highlighted by reciprocal transplant experiments demonstrating that fecal microbiota from young donors, when transferred into aged recipients, reverses aging-associated differences in peripheral and hippocampal immune responses, restores the hippocampal metabolome and transcriptome to more youthful profiles, reduces pro-inflammatory microglial gene expression, and attenuates selective age-associated cognitive impairments (Boehme et al., 2021). The fact that the aged brain can be partially rescued by a young microbiome, without any direct intervention in the central nervous system, reinforces the view that the microbiome may represent a modifiable contributor to age-dependent brain vulnerability rather than simply a biomarker of age-related decline.

## 3 Mechanistic pathways linking diet, the gut microbiota, and brain function

Diet is widely recognized as a central environmental determinant shaping the composition, diversity, and metabolic activity of the gut microbiota. Through the provision of nutrients and bioactive compounds, dietary patterns exert selective pressures that influence microbial community structure and functional capacity. Experimental studies demonstrate that dietary interventions can rapidly alter gut microbial composition and metabolic activity (David et al., 2014). These diet-driven shifts in microbial ecology modulate the production of key metabolites, regulate immune signaling pathways, and influence neurobiological processes underlying brain function (Morais et al., 2021). As such, diet represents a key upstream factor linking the gut microbiota to systemic physiology and cognitive health. The following sections outline the principal mechanistic pathways linking diet, gut microbiota metabolism, immune signaling, and brain function, highlighting how diet-driven microbial metabolites may influence cognitive health.

### 3.1 Diet-driven modulation of gut microbiota composition and function

The composition and functional capacity of the gut microbiota are influenced by habitual dietary intake. Different dietary components can selectively promote or suppress the growth of specific microbial taxa, thereby shaping microbial diversity and metabolic activity. In particular, the interplay between dietary fiber, polyphenols, and fats has emerged as a critical determinant of microbial community structure, with important implications for host metabolism and brain health (Graf et al., 2015).

#### 3.1.1 Dietary Fiber

Dietary fiber plays a key role in shaping the structure and metabolic activity of the gut microbiota. Unlike digestible carbohydrates, many dietary fibers escape enzymatic digestion in the small intestine and reach the colon, where they serve as fermentable substrates for specific microbial taxa. Through microbial fermentation, these fibers promote the proliferation of beneficial commensal bacteria, particularly members of the genera *Bifidobacterium* and *Lactobacillus*, as well as several butyrate-producing taxa within the families *Lachnospiraceae* and Ruminococcaceae (Graf et al., 2015). Emerging evidence suggests that SCFAs produced as a result of dietary fiber fermentation by the gut microbiota can influence central nervous system function through multiple mechanisms, including immune modulation, vagal signaling, and epigenetic regulation within the brain (Koh et al., 2016).

#### 3.1.2 Polyphenols

Polyphenols constitute a diverse group of plant-derived bioactive compounds that exert significant modulatory effects on gut microbial composition and metabolic activity. Because a substantial proportion of dietary polyphenols is poorly absorbed in the small intestine, many of these compounds reach the colon, where they undergo extensive biotransformation by the gut microbiota. This bidirectional interaction is particularly important: intestinal microbes convert complex polyphenols into smaller, more bioavailable metabolites, while polyphenols themselves exert selective pressures on microbial community structure (Cardona et al., 2013).

Preclinical and human studies have demonstrated that polyphenol-rich foods, including fruits, berries, cocoa, and tea, can selectively promote the growth of beneficial microbial taxa such as *Bifidobacterium*, *Lactobacillus*, and *Akkermansia muciniphila*, while suppressing the proliferation of potentially pathogenic microorganisms (Roopchand et al., 2015; Vendrame et al., 2011). These microbiota-associated shifts are frequently accompanied by improvements in metabolic and inflammatory profiles, highlighting the capacity of polyphenols to modulate gut microbial homeostasis and associated host responses.

Beyond altering microbial composition, microbiota-derived polyphenol metabolites, such as phenolic acids and urolithins, can influence several pathways involved in gut–brain communication. These metabolites have been shown to attenuate oxidative stress, regulate inflammatory signaling, and interact with neuronal pathways relevant to cognitive function and neuroprotection (Del Rio et al., 2013; Vauzour et al., 2008). Through these mechanisms, dietary polyphenols contribute to the establishment of a gut microbial environment that supports systemic metabolic health and may contribute to cognitive resilience.

#### 3.1.3 Dietary fats

Dietary fats represent an important determinant influencing gut microbial composition and metabolic activity, with effects largely determined by fatty acid type rather than total fat intake. Diets rich in saturated fats, characteristic of Western dietary patterns, have been consistently associated with reduced microbial diversity and the expansion of pro-inflammatory, bile-tolerant taxa such as *Bilophila wadsworthia* (Devkota et al., 2012; Hildebrandt et al., 2009; Natividad et al., 2018). These microbiota alterations can disrupt intestinal barrier integrity and promote metabolic endotoxemia, a condition characterized by increased circulating lipopolysaccharides (LPS) that trigger chronic low-grade systemic inflammation (Cani et al., 2007).

In contrast, unsaturated fats, particularly omega-3 polyunsaturated fatty acids (PUFAs), have been associated with beneficial shifts in the gut microbial ecosystem. Experimental evidence indicates that omega-3 fatty acids can increase the abundance of beneficial taxa, including *Akkermansia muciniphila*, while attenuating inflammatory responses linked to metabolic dysfunction (Caesar et al., 2012). These microbiota-associated changes may contribute to improved metabolic regulation and reduced inflammatory signaling.

Moreover, diets high in saturated fats are also linked to neuroinflammatory processes, including microglial activation and increased inflammatory signaling within the central nervous system (Bruce-Keller et al., 2015). Collectively, these findings highlight that the quality of dietary fats, rather than fat quantity alone, plays a critical role in shaping gut microbial ecology and influencing downstream inflammatory and neurobiological pathways relevant to metabolic and cognitive health.

### 3.2 Microbiota-derived metabolites as mediators of gut–brain communication

The gut microbiota functions as a “virtual endocrine organ,” converting dietary substrates into a diverse array of bioactive metabolites that serve as key mediators of the microbiota–gut–brain axis. Through microbial fermentation of dietary components and transformation of host-derived substrates, intestinal microbes generate signaling molecules capable of influencing host physiology beyond the gastrointestinal tract. Among these, short-chain fatty acids (SCFAs), tryptophan-derived metabolites, and microbially modified bile acids represent three major metabolic classes through which gut microbial activity influences host metabolic, immune, and neurophysiological processes.

These metabolites act as chemical messengers that regulate multiple host pathways, including immune, endocrine, and neural signaling. By modulating intestinal barrier integrity, shaping systemic and neuroinflammatory responses, and interacting with receptors expressed in both peripheral tissues and the central nervous system, microbiota-derived metabolites play a critical role in influencing neurobiological processes relevant to cognitive function and brain health (Dalile et al., 2019).

#### 3.2.1 Short-chain fatty acids

Among microbiota-derived metabolites, SCFAs are among the most influential classes due to their wide-ranging metabolic, immunological, and neurobiological effects within the microbiota–gut–brain axis. In addition to serving as a major energy source for colonocytes, SCFAs can enter the systemic circulation and influence host metabolism and immune responses through their interaction with G protein-coupled receptors (GPCRs), particularly GPR41 (FFAR3) and GPR43 (FFAR2) (Brown et al., 2003).

Beyond their barrier-regulating functions, SCFAs act as epigenetic modulators. Butyrate functions as a histone deacetylase (HDAC) inhibitor, promoting chromatin relaxation and influencing the expression of neurotrophic factors, including brain-derived neurotrophic factor (BDNF), which plays a central role in neuronal survival, synaptic plasticity, and cognitive function (Stilling et al., 2016).

Furthermore, SCFAs contribute to the regulation of neuroimmune processes within the central nervous system. Experimental studies have demonstrated that microbiota-derived metabolites influence the maturation and function of microglia, the resident immune cells of the brain, thereby shaping neuroinflammatory responses and neuronal homeostasis (Erny et al., 2015).

Within the context of aging, reductions in SCFA production, often associated with age-related alterations in gut microbiota composition and a decline in butyrate-producing bacteria such as *Faecalibacterium prausnitzii*, have been linked to increased intestinal permeability, heightened neuroinflammation, and cognitive decline (Claesson et al., 2012).

Collectively, these findings suggest that SCFAs are not merely microbial fermentation by-products but rather key molecular mediators that contribute to neurobiological resilience and help maintain central nervous system homeostasis across the lifespan. SCFAs exemplify how microbial metabolic outputs can shape host immune, metabolic, and neural pathways, highlighting the broader role of microbiota-derived metabolites in gut–brain communication.

#### 3.2.2 Tryptophan metabolism and indole-derived signaling

Tryptophan (Trp), an essential dietary amino acid, represents a major biochemical axis through which the gut microbiota can influence host physiology and brain function. Within the host–microbiota system, Trp metabolism proceeds through three interconnected routes: the kynurenine pathway (under host enzymatic control), serotonin biosynthesis in enterochromaffin and neuronal cells, and microbial conversion into indole derivatives. Together, these routes yield a broad spectrum of bioactive metabolites that converge on immune, metabolic, and neural signaling, thereby enabling bidirectional communication along the microbiota–gut–brain axis (Cervantes-Barragan et al., 2017; Rothhammer et al., 2016; Yano et al., 2015).

The kynurenine pathway (KP) represents the principal route of tryptophan degradation in mammals. It is initiated by the heme-dependent dioxygenases indoleamine-2,3-dioxygenase (IDO1/IDO2) and tryptophan-2,3-dioxygenase (TDO), which catalyze the oxidative cleavage of tryptophan to N-formylkynurenine, followed by conversion into kynurenine (Schwarcz et al., 2012). Downstream, KP metabolism yields several neuroactive metabolites that differentially shape neuronal and immune signaling. Among these, kynurenic acid (KYNA) and quinolinic acid (QUIN) represent functionally opposing metabolites with significant implications for brain physiology: KYNA acts predominantly as a neuroprotective antagonist of ionotropic glutamate receptors, particularly at the glycine co-agonist site of the N-methyl-D-aspartate (NMDA) receptor, whereas QUIN functions as a potent NMDA receptor agonist capable of inducing excitotoxic neuronal injury and promoting neuroinflammatory responses (Schwarcz et al., 2012). The balance between KYNA and QUIN production is tightly governed by cell-type–specific metabolism within astrocytes and microglia, underscoring the central role of glial cells in regulating KP activity in the brain. Consequently, dysregulation of KP metabolism perturbs neuroimmune homeostasis and has been implicated in a range of neurological and neurodegenerative disorders, including Alzheimer’s disease, Parkinson’s disease, and major depressive disorder (Guillemin et al., 2005; Minhas et al., 2024; Stone and Darlington, 2013).

Alongside the kynurenine pathway, tryptophan also serves as a precursor for serotonin (5-hydroxytryptamine, 5-HT), a key signaling molecule involved in gastrointestinal function, immune modulation, and neuroendocrine communication (Cervantes-Barragan et al., 2017). Serotonin signaling is highly compartmentalized. Although 5-HT functions as an important neurotransmitter in the central nervous system (CNS), approximately 95% of the body’s serotonin resides in the gastrointestinal tract, where it is produced predominantly by enterochromaffin (EC) cells located in the intestinal mucosa, with a smaller contribution from enteric serotonergic neurons (Berger et al., 2009; Gershon and Tack, 2007). Because peripheral serotonin does not readily cross the blood–brain barrier under physiological conditions, its influence on brain function occurs primarily through indirect mechanisms, including vagal signaling, immune–endocrine interactions, and vascular or metabolic pathways (Berger et al., 2009).

Serotonin biosynthesis within the intestinal mucosa is initiated by the rate-limiting enzyme tryptophan hydroxylase-1 (TPH1), which catalyzes the conversion of tryptophan to 5-hydroxytryptophan (5-HTP), followed by decarboxylation to generate serotonin. Newly synthesized serotonin is released into the intestinal lumen and lamina propria, after which it is rapidly sequestered by circulating platelets, the principal peripheral reservoir of 5-HT. In contrast, central serotonin is synthesized by neurons through the isoform tryptophan hydroxylase-2 (TPH2). The restrictive properties of the blood–brain barrier maintain a functional separation between peripheral and central serotonergic pools, thereby preserving the distinct physiological roles of serotonin signaling in the gut and the brain (Berger et al., 2009).

Accumulating evidence indicates that the gut microbiota exert substantial regulatory control over peripheral serotonin production. Experimental studies in germ-free and recolonized animals have demonstrated that spore-forming commensal bacteria stimulate serotonin biosynthesis in enterochromaffin cells and restore gastrointestinal motility and platelet function, providing direct evidence that microbial communities regulate host serotonergic tone (Yano et al., 2015). In addition, microbial metabolites such as short-chain fatty acids (SCFAs) enhance mucosal Tph1 expression and stimulate EC-cell activity, identifying a metabolite-dependent pathway through which intestinal microbes modulate host serotonin synthesis (Reigstad et al., 2014).

Gut microbes can also produce tryptophan-derived molecules that directly interact with serotonergic signaling pathways. A well-characterized example is tryptamine, a microbial monoamine capable of activating epithelial 5-HT₄ receptors. Activation of this pathway increases intestinal fluid secretion and accelerates gut transit, illustrating how microbial metabolites can mimic or amplify host serotonergic regulation within the gastrointestinal tract (Bhattarai et al., 2018). Collectively, these observations indicate that microbial regulation of serotonin involves both enterochromaffin cell biosynthesis and serotonin-like microbial metabolites, thereby contributing to microbiota–gut–brain communication and intersecting with additional tryptophan-derived signaling pathways discussed in the following section.

Beyond host-mediated kynurenine and serotonin pathways, intestinal microbes convert a portion of dietary tryptophan into a diverse group of indole-derived metabolites that function as signaling molecules within the host. These metabolites, including indole, indole-3-aldehyde (IAld), indole-3-propionic acid (IPA), and indole-3-acetic acid (IAA), represent an additional branch of tryptophan metabolism that links microbial activity to host epithelial and immune regulation (Li et al., 2021; Roager and Licht, 2018).

The physiological effects of these microbial metabolites are largely mediated through host receptors, most notably the aryl hydrocarbon receptor (AhR) and the pregnane X receptor (PXR) (Li et al., 2021). For instance, IAld produced by *Lactobacillus* species activates AhR signaling and promotes interleukin-22 (IL-22)-dependent mucosal protection, thereby enhancing resistance to intestinal pathogens (Zelante et al., 2013). In contrast, IPA functions as a ligand of PXR, suppressing epithelial inflammatory signaling and promoting the expression of tight-junction genes, which collectively reinforce intestinal barrier integrity (Venkatesh et al., 2014). Indole itself can also contribute to epithelial homeostasis by increasing transepithelial resistance and attenuating inflammatory pathways in intestinal epithelial cells (Bansal et al., 2010).

Importantly, the influence of microbial indole metabolites extends beyond the intestinal environment. Experimental evidence indicates that microbiota-derived tryptophan metabolites acting through AhR signaling can regulate astrocyte activity and limit central nervous system inflammation, highlighting a mechanistic pathway through which microbial metabolism contributes to neuroimmune homeostasis along the microbiota–gut–brain axis (Rothhammer et al., 2016).

**Figure 2:**
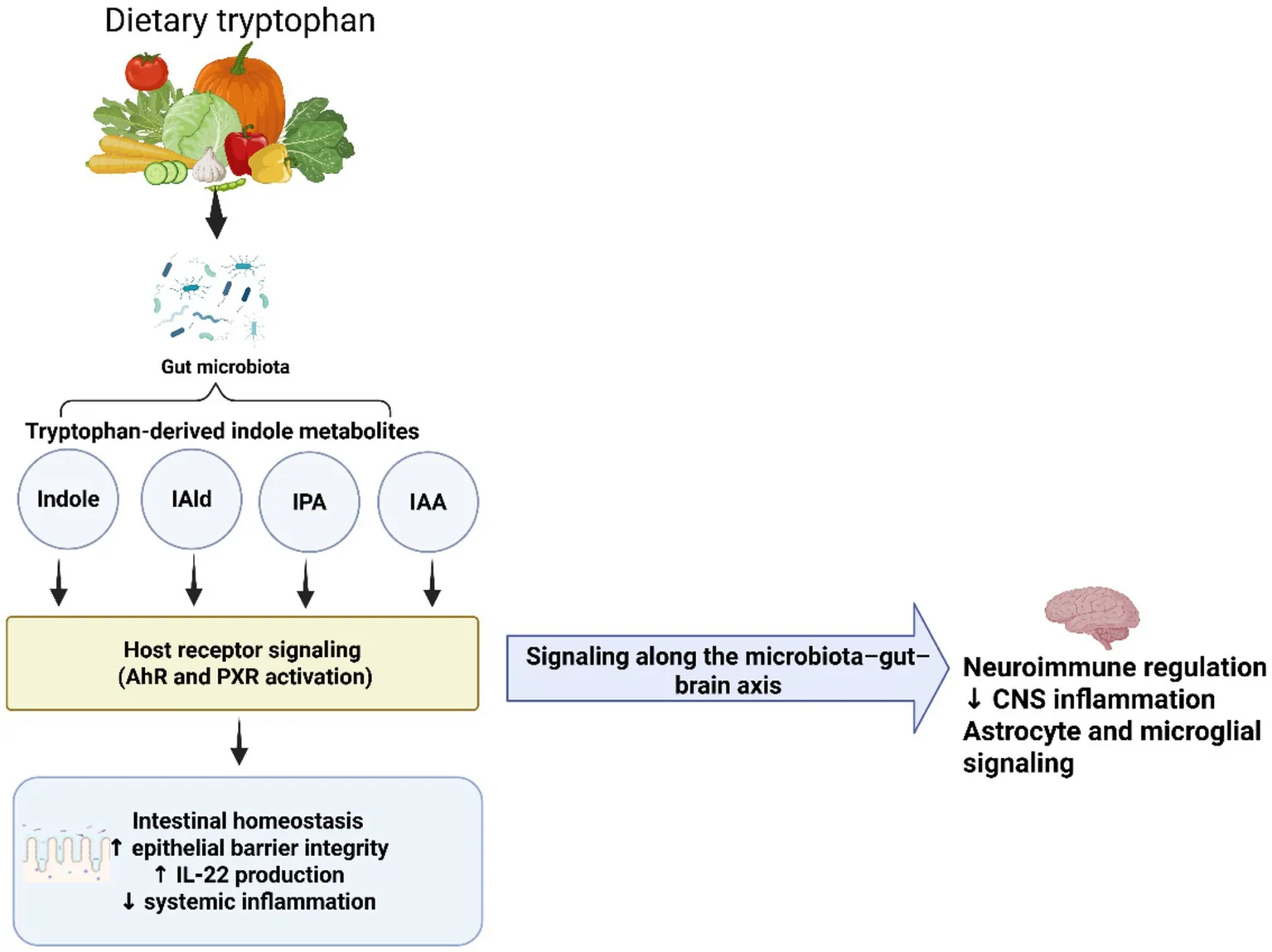
Key mechanistic pathways linking microbial tryptophan metabolism to host signaling and neuroimmune regulation. Dietary tryptophan is metabolized by the gut microbiota into indole derivatives, including indole, indole-3-aldehyde (IAld), indole-3-propionic acid (IPA), and indole-3-acetic acid (IAA). These metabolites activate host receptors such as the aryl hydrocarbon receptor (AhR) and pregnane X receptor (PXR), contributing to intestinal barrier integrity, increased interleukin-22 (IL-22) production, and reduced inflammatory signaling. Through signaling along the microbiota–gut–brain axis, these pathways contribute to neuroimmune regulation and modulation of central nervous system inflammation. Other microbiota-derived metabolites, including bile acids, also contribute to host signaling along the microbiota–gut–brain axis and play roles in metabolic, immune, and neurophysiological regulation. Created in BioRender. Jarfan, M. (2026). https://BioRender.com/sondve8.

Collectively, these pathways highlight tryptophan metabolism as a central integrative hub linking microbial activity to host immune, metabolic, and neurophysiological regulation along the microbiota–gut–brain axis. The major microbial indole metabolites and their host signaling pathways are summarized in Table 1, while Figure 2 illustrates the overall microbial metabolism of dietary tryptophan.

**Table 1:** Representative microbial indole metabolites, host receptors, and principal physiological effects.

Metabolite	Microbial origin	Host receptor	Physiological effects	Key reference
Indole	*Escherichia coli* and *Bacteroides* spp.	AhR and epithelial signaling	Enhances epithelial barrier integrity and suppresses inflammatory signaling	Bansal et al. (2010) and Li et al. (2021)
Indole-3-aldehyde (IAld)	*Lactobacillus* spp.	AhR	Promotes IL-22 production and mucosal immune protection	Zelante et al. (2013)
Indole-3-propionic acid (IPA)	Anaerobic gut microbiota	PXR	Suppresses epithelial inflammation and strengthens gut barrier function	Venkatesh et al. (2014)
Indole-3-acetic acid (IAA)	Gut microbiota	AhR	Modulates mucosal immunity and intestinal homeostasis	Li et al. (2021)
Indole derivatives (general)	Diverse gut microbiota	AhR signaling (astrocytes)	Modulates CNS inflammation and astrocyte-mediated neuroimmune signaling	Rothhammer et al. (2016)

Rows summarize representative microbial tryptophan-derived indole metabolites and their principal host signaling pathways along the microbiota–gut–brain axis (AhR = aryl hydrocarbon receptor; PXR = pregnane X receptor).

#### 3.2.3 Microbial transformation of bile acids

Bile acids represent an important interface between host metabolism and microbial activity within the intestine. They are synthesized from cholesterol in the liver to generate the primary bile acids cholic acid (CA) and chenodeoxycholic acid (CDCA). Before biliary secretion, these primary bile acids are conjugated with glycine or taurine, which increases their solubility and facilitates participation in enterohepatic circulation (Wahlström et al., 2016). Following their release into the intestinal lumen, most bile acids are reabsorbed in the distal ileum; however, a fraction reaches the colon, where they undergo microbial transformations (Chiang and Ferrell, 2020; Wahlström et al., 2016). These reactions, including deconjugation, dehydroxylation, dehydrogenation, and epimerization, convert primary bile acids into secondary bile acids such as deoxycholic acid (DCA) and lithocholic acid (LCA). This process expands the chemical diversity of the bile acid pool and alters its signaling capacity (Guzior and Quinn, 2021; Ridlon et al., 2006).

Microbial conversion of bile acids is initiated primarily by bile salt hydrolases (BSHs), a widely distributed family of enzymes among intestinal bacteria, including *Lactobacillus*, *Bifidobacterium*, *Bacteroides*, and *Clostridium* species. BSH-mediated deconjugation represents the principal gateway reaction in microbial bile acid metabolism, generating substrates for downstream transformations. Following deconjugation, a restricted group of anaerobic bacteria, most notably members of the genus *Clostridium*, carry out 7α-dehydroxylation reactions that produce secondary bile acids such as DCA and LCA. Recent mechanistic studies have clarified the enzymatic pathways and genetic organization underlying these transformations, demonstrating how gut microbes actively regulate the composition of the bile acid pool (Bourgin et al., 2021; Funabashi et al., 2020; Joyce and Gahan, 2016; Ridlon et al., 2006).

Beyond their classical role in lipid digestion, bile acids also function as endocrine-like signaling molecules that regulate multiple aspects of host physiology. Two major receptors mediate these effects: the nuclear farnesoid X receptor (FXR) and the membrane G protein–coupled receptor TGR5 (GPBAR1). Activation of intestinal FXR induces fibroblast growth factor 19 (FGF19), which provides negative feedback on hepatic bile acid synthesis and contributes to the regulation of glucose and lipid homeostasis. In parallel, TGR5 signaling influences energy expenditure, innate immune responses, and intestinal barrier integrity. Through these receptor-dependent pathways, microbial remodeling of bile acids links gut microbial activity with systemic metabolic and inflammatory regulation (Chiang and Ferrell, 2020; Fiorucci and Distrutti, 2015; Wahlström et al., 2016).

Growing evidence indicates that bile acid metabolism also contributes to microbiota–gut–brain axis communication. Alterations in circulating bile acid profiles have been associated with metabolic dysfunction, systemic inflammation, and several neurodegenerative disorders. Experimental and clinical studies suggest that bile acid-mediated signaling can influence neuroinflammatory pathways, modulate microglial activation, and affect central nervous system function. Dysregulated bile acid composition has been documented in neurological conditions, including Alzheimer’s disease and Parkinson’s disease, suggesting that microbial modulation of the bile acid pool may contribute to neurodegenerative processes through interconnected metabolic, immunological, and barrier-related mechanisms (Baloni et al., 2020; MahmoudianDehkordi et al., 2019; Nho et al., 2019).

Microbial transformation of bile acids broadens the spectrum of microbiota-derived metabolites linking intestinal microbial ecology with host physiology. Through FXR- and TGR5-mediated signaling pathways, these metabolites connect gut microbial activity with systemic metabolic regulation, immune homeostasis, and neurophysiological processes along the microbiota–gut–brain axis.

These metabolite classes illustrate how gut microbial metabolism translates dietary inputs into molecular signals that regulate host metabolic, immune, and neurophysiological functions. In this way, microbial metabolites act as central mediators linking diet, microbiota composition, and brain function along the microbiota–gut–brain axis.

### 3.3 Microbial metabolites and immune signaling in the gut–brain axis

The gastrointestinal tract, through gut-associated lymphoid tissue (GALT), is considered one of the most immunologically active interfaces in the human body, enabling continuous crosstalk between commensal microbes and both innate and adaptive host defense systems. Within this environment, host–microbe interactions help shape mucosal and systemic immune responses that influence brain function (Kasarello et al., 2023; Morais et al., 2021).

Under physiological conditions, intestinal barrier integrity is maintained by epithelial tight junctions and mucosal defenses that restrict the translocation of luminal antigens. However, disruption of gut microbial composition (dysbiosis) compromises these protective mechanisms, leading to increased intestinal permeability and facilitating the translocation of microbial components, particularly lipopolysaccharide (LPS), into the circulation, where LPS activates Toll-like receptor 4 (TLR4)/CD14-dependent signaling pathways, inducing metabolic endotoxemia and triggering systemic inflammatory responses (Cani et al., 2007).

In parallel, microbiota-derived metabolites play central roles in maintaining mucosal immune homeostasis. SCFAs, particularly butyrate, promote regulatory T-cell (Treg) differentiation via histone deacetylase inhibition and epigenetic regulation of the Foxp3 locus, thereby supporting an anti-inflammatory mucosal environment (Furusawa et al., 2013). Tryptophan-derived indole metabolites activate aryl hydrocarbon receptor (AhR) signaling pathways, stimulating interleukin-22 (IL-22)-mediated responses that reinforce epithelial barrier integrity and mucosal immunity (Zelante et al., 2013). Additionally, microbially modified bile acid metabolites, such as 3-oxoLCA and isoalloLCA, regulate T-helper cell differentiation by suppressing pro-inflammatory Th17 responses and promoting Treg induction, thereby maintaining immune balance at the intestinal interface (Hang et al., 2019).

When these immune–microbial regulatory mechanisms are disrupted by dysbiosis, elevated circulating cytokines, including interleukin-6 (IL-6), tumor necrosis factor-alpha (TNF-*α*), and interleukin-1β (IL-1β), can sustain chronic low-grade inflammation. With aging, this persistent inflammatory state evolves into “inflammaging,” a hallmark of biological aging that has been associated with neuroinflammation and cognitive decline (Chakrabarti et al., 2022; Franceschi et al., 2018).

Peripheral inflammatory signals extend to the central nervous system, where they influence microglial activation and neuroimmune responses. Sustained exposure to circulating cytokines and microbiota-derived signals drives microglia toward pro-inflammatory phenotypes, amplifying neuroinflammatory processes and impairing synaptic plasticity and neuronal function (Erny et al., 2015; Mosher and Wyss-Coray, 2014).

Collectively, these interconnected pathways, spanning GALT-mediated immune signaling, intestinal barrier regulation, microbiota-derived metabolite activity, systemic cytokine dynamics, inflammaging, and neuroimmune activation, are increasingly implicated in neuroinflammation and may contribute to cognitive decline during aging (Chakrabarti et al., 2022).

### 3.4 Neurobiological mechanisms linking the gut microbiota to brain function

Beyond immune-mediated pathways, the gut microbiota influences brain function through a combination of neural, endocrine, metabolic, and barrier-related mechanisms that converge to regulate neuronal activity, synaptic plasticity, and cognitive processes (Cryan and Dinan, 2012; Mayer et al., 2014).

#### 3.4.1 Neural signaling

The vagus nerve represents a primary bidirectional communication pathway within the microbiota–gut–brain axis (Bonaz et al., 2018). Microbiota-derived metabolites and gut-derived signaling molecules can activate vagal afferents either directly or via specialized enteroendocrine cells and peptide mediators, thereby transmitting signals to brain regions involved in emotional regulation, stress responsiveness, and cognition (Dockray, 2013; Kaelberer et al., 2018). Experimental evidence indicates that disruption of vagal signaling attenuates microbiota-dependent behavioral and neurochemical effects in preclinical models, underscoring the central role of vagal pathways in gut–brain communication (Bravo et al., 2011).

#### 3.4.2 Endocrine signaling

The gut microbiota influences neuroendocrine function in part through its effects on the hypothalamic–pituitary–adrenal (HPA) axis. Germ-free animals show exaggerated corticosterone and adrenocorticotropic hormone (ACTH) responses, which can be normalized following microbial colonization, pointing to a role for the microbiota in shaping stress-related neuroendocrine activity (Dinan and Cryan, 2017; Sudo et al., 2004). Dysregulation of the HPA axis has been associated with altered stress reactivity and linked to neuroinflammatory processes that may contribute to cognitive dysfunction (Dinan and Cryan, 2017; Lupien et al., 2009). Experimental studies further support the involvement of the gut microbiota in modulating stress-related neuroendocrine pathways and behavior (De Palma et al., 2015). However, the extent to which these mechanisms translate to human physiology remains unclear and is likely context-dependent.

#### 3.4.3 Neurotransmitter and tryptophan-related signaling

The gut microbiota contributes to neurochemical signaling through both the production of neurotransmitter-like compounds and the modulation of key metabolic precursors (Dalile et al., 2019). Certain microbial taxa have been implicated in modulating gamma-aminobutyric acid (GABA) signaling and, to a lesser extent, dopaminergic pathways, supporting a role in shaping host neurochemical communication through microbiota–host interactions (Strandwitz, 2018). In parallel, microbial modulation of tryptophan metabolism, particularly via the generation of indole derivatives that activate aryl hydrocarbon receptor (AhR) signaling and induce interleukin-22 (IL-22)–mediated responses, connects microbial metabolism with mucosal immune activity and gut–brain communication pathways via immune, vagal, and metabolic routes (Agus et al., 2018). Evidence from microbiota transfer studies further supports a functional link between microbial composition and brain-related outcomes (Sharon et al., 2016). Although peripheral serotonin does not cross the blood–brain barrier, these pathways offer a biologically plausible route through which microbial metabolism may influence central nervous system function.

#### 3.4.4 Synaptic plasticity and neurotrophic signaling

Microbiota-derived metabolites, particularly short-chain fatty acids such as butyrate, may influence synaptic plasticity, at least in part, through epigenetic regulation. As a histone deacetylase inhibitor, butyrate has been shown to promote histone acetylation at promoters of plasticity-related genes, providing a plausible mechanistic link between microbial metabolism and transcriptional processes associated with neuroplasticity (Dalile et al., 2019; Stilling et al., 2016). This pathway has been proposed to be indirectly associated with the regulation of brain-derived neurotrophic factor (BDNF), although current evidence remains largely preclinical and translational. While these findings provide mechanistic insights into microbiota–brain interactions, their relevance to human physiology remains to be established.

#### 3.4.5 Blood–brain barrier regulation

The gut microbiota has been implicated in maintaining blood–brain barrier (BBB) integrity. In germ-free mouse models, the absence of a gut microbiota increases BBB permeability and reduces the expression of tight junction proteins such as claudin-5 and occludin, whereas microbial colonization restores barrier structure and function (Braniste et al., 2014). These findings suggest that microbiota-dependent regulation of barrier integrity may contribute to central nervous system homeostasis by modulating the access of peripheral immune signals to the brain. Preclinical evidence further indicates that BBB disruption can permit the entry of circulating inflammatory mediators into the central nervous system, potentially amplifying neuroinflammatory processes; however, direct evidence for comparable processes in humans remains limited.

Collectively, these findings suggest that microbiota-driven regulation of synaptic plasticity and blood–brain barrier integrity may represent complementary pathways linking gut microbial activity to brain function. Despite increasingly detailed mechanistic insights, causal relationships in humans remain largely unestablished and require validation in well-controlled clinical settings.

Taken together, SCFAs, tryptophan-derived metabolites, and microbially modified bile acids should not be viewed as entirely independent signaling pathways. Although these metabolite classes act through distinct receptors and molecular mechanisms, each has been implicated in aspects of immune regulation, inflammatory signaling, and gut–brain communication (Baloni et al., 2020; Erny et al., 2015; Nho et al., 2019; Rothhammer et al., 2016; Zelante et al., 2013). These shared biological effects suggest that multiple microbiota-derived metabolites converge on common processes relevant to neuroinflammation, neuroimmune regulation, and cognitive aging.

Importantly, the strength of evidence differs across pathways. SCFAs currently possess the most extensive mechanistic and translational evidence, whereas evidence for tryptophan-derived metabolites and bile acid signaling remains comparatively less developed in terms of translational and interventional evidence, particularly regarding direct causal links to cognitive outcomes in humans.

These observations suggest that future microbiome-informed interventions may benefit from targeting shared biological processes, including microbial metabolite production, intestinal barrier function, immune regulation, and neuroimmune signaling, rather than focusing exclusively on individual biomarkers or specific metabolite classes.

### 3.5 Clinical evidence linking diet, the gut microbiota, and cognitive function

#### 3.5.1 Observational evidence

Epidemiological studies have reported associations between dietary patterns and cognitive health, with adherence to the Mediterranean diet and the Mediterranean–DASH Intervention for Neurodegenerative Delay (MIND) dietary patterns linked to slower cognitive decline and, in some cohorts, lower risk of neurodegenerative outcomes (Morris et al., 2015; Scarmeas et al., 2006). These patterns are generally characterized by higher intake of plant-based foods, including foods rich in fiber, polyphenols, and unsaturated fats, and are often interpreted as supporting microbial diversity and metabolite production, although such interpretations remain indirect.

However, these interpretations require careful consideration. Observational evidence in this domain is inherently limited in its ability to support causal inference. Residual confounding, correlated health behaviors, reverse causation, and measurement error in dietary assessment remain central methodological challenges in nutritional epidemiology research (Ioannidis, 2018; Maki et al., 2014; Satija et al., 2015). While these studies provide important population-level insights, the observed associations may reflect broader health-related behavioral phenotypes rather than diet-specific biological effects. As a result, attributing cognitive outcomes to individual dietary components risks overstating mechanistic specificity within inherently complex and interdependent exposures.

#### 3.5.2 Diet–microbiota interactions

Diet is widely regarded as a key modifiable determinant of gut microbial composition; however, the magnitude, stability, and functional significance of this relationship remain insufficiently resolved. While plant-based and fiber-rich dietary patterns have been associated with increased abundance of short-chain fatty acid–producing taxa, such as *Faecalibacterium* and *Roseburia*, to date, these associations have not been consistently reproduced across populations or study designs and appear to depend on baseline microbiome configuration, host genetics, and environmental context (Johnson et al., 2019; Rothschild et al., 2018; Wu et al., 2011).

Importantly, the functional consequences of such compositional shifts remain poorly delineated. Frequently cited downstream effects, including potential modulation of barrier integrity and inflammatory signaling, are largely inferred from experimental and translational models rather than directly demonstrated in human populations, although they provide biologically plausible hypotheses (Koh et al., 2016). This reliance on indirect inference thus raises fundamental concerns regarding external validity and mechanistic translation. Moreover, the link between these microbial processes and domain-specific cognitive outcomes, such as executive function, memory, and processing speed, remains insufficiently characterized (Mayer et al., 2015).

At a broader level, the field remains constrained by limited taxonomic and functional specificity. Similar microbial signatures are repeatedly associated with a wide range of health outcomes, including metabolic, inflammatory, and neurocognitive phenotypes. While informative at an ecological level, this lack of discriminatory resolution suggests that current frameworks remain insufficient for delineating causal pathways. Consequently, many reported diet–microbiome associations are more likely to reflect general ecological restructuring rather than discrete, mechanistically coherent effects (Lynch and Pedersen, 2016).

#### 3.5.3 Intervention studies

Randomized controlled trials (RCTs) are generally regarded as providing higher-level evidence; however, their interpretability in this field remains constrained by methodological limitations and underlying biological variability. Dietary interventions based on Mediterranean-style patterns, as well as microbiome-targeted approaches including probiotic and prebiotic supplementation, have reported modest improvements in selected cognitive, neurobehavioral, or stress-related outcomes in some trials (Allen et al., 2016; Ngandu et al., 2015; Valls-Pedret et al., 2015). However, effects are generally limited, domain-specific, and not consistently reproduced across studies.

For example, the Prevención con Dieta Mediterránea (PREDIMED) study reported improvements in composite cognitive scores; however, such effects were not consistently observed across individual neuropsychological domains. The observed effects were largely confined to composite z-score outcomes, raising concerns regarding clinical interpretability and outcome specificity, particularly given the *post hoc* design, selective attrition, and population-specific characteristics of the cohort (Valls-Pedret et al., 2015). In contrast, the more recent 3-year MIND diet randomized trial found no significant between-group differences in global cognition or brain MRI outcomes despite good adherence and weight loss in both intervention arms (Barnes et al., 2023).

Similarly, microbiome-targeted interventions using probiotic and prebiotic strategies have yielded heterogeneous and, in some cases, methodologically constrained findings rather than a consistent pattern of cognitive benefit. For example, one trial reported improvements in MMSE scores following probiotic supplementation in patients with Alzheimer’s disease; however, the study was characterized by a small sample size and reliance on a single cognitive outcome (Akbari et al., 2016). In another study in community-dwelling older adults, probiotic supplementation was associated with improvements in selected cognitive domains, such as mental flexibility, alongside changes in gut microbiota composition; however, these effects remain domain-specific and not uniformly observed across broader cognitive measures (Kim et al., 2021). In addition, some trials suggest that potential benefits may be more pronounced in subgroups with baseline cognitive impairment, rather than across the general population, further limiting generalizability. For example, randomized trials have stratified participants by cognitive status and reported microbiome and cognitive changes within cognitively impaired subgroups following probiotic supplementation, although direct between-group comparisons remain limited (Aljumaah et al., 2022).

Prebiotic interventions provide an additional illustration of this interpretive complexity. For instance, galactooligosaccharide (GOS) supplementation has been shown to reduce waking cortisol responses and modify attentional bias toward emotional stimuli in healthy individuals (Schmidt et al., 2015). However, these outcomes are more appropriately interpreted as neuroendocrine and affective-processing measures rather than direct indicators of core cognitive domains such as memory, executive function, or processing speed. Similarly, in the MedDairy randomized crossover trial, improvements were observed in processing speed and mood-related outcomes, but not in attention, memory, planning, or dementia-risk measures, suggesting that intervention effects may emerge in selective or proxy endpoints rather than across a coherent cognitive profile (Wade et al., 2020).

Finally, evidence from vitamin and mineral supplementation trials, arguably representing more controlled and precisely defined nutritional interventions, has demonstrated largely null or clinically insignificant effects on cognitive outcomes. Several randomized controlled trials have reported limited or no cognitive benefits following supplementation with vitamins or micronutrients, including studies of B vitamins and antioxidant supplementation (De Jager et al., 2012; Ford et al., 2010). Cochrane systematic reviews of randomized controlled trials report no convincing evidence that such interventions prevent cognitive decline or dementia, with most studies limited by short duration, suboptimal cognitive outcome measures, and heterogeneous findings (Rutjes et al., 2018).

This heterogeneity likely arises from both methodological variation and intrinsic biological complexity. Many trials remain relatively short in duration, outcome-specific in design, and vulnerable to differences in intervention composition, adherence, comparator quality, and statistical power. Moreover, inter-individual variation in baseline microbiome configuration may influence responsiveness to dietary and microbiome-targeted interventions, raising the possibility that modest or null group-level effects may obscure responder-specific benefits (Zmora et al., 2019). Taken together, current evidence does suggest potential benefit; however, these remain context-dependent and inconsistently expressed, and do not yet provide a sufficiently consistent or mechanistically coherent basis for clinically generalizable causal claims for microbiome-mediated cognitive enhancement.

#### 3.5.4 Limitations

The evidence base is characterized not only by heterogeneity but also by structural and methodological fragmentation. Differences in microbiome profiling techniques, sequencing depth, analytical pipelines, and dietary assessment methods introduce substantial variability and limit cross-study comparability (Knight et al., 2018). The relative scarcity of longitudinal human studies further constrains the ability to capture temporal dynamics and to infer causality.

A key limitation of research conducted thus far is a disconnect between mechanistic and clinical evidence. While preclinical models provide increasingly detailed insights into microbiota–brain interactions, their translation to human physiology remains unclear (Kundu et al., 2017; Muller et al., 2020). As a result, the field lacks integrative frameworks capable of reconciling mechanistic pathways with clinically meaningful, human-relevant outcomes.

In addition, many studies have been conducted in relatively homogeneous populations, limiting generalizability across diverse demographic and clinical contexts. The bidirectional nature of the gut–brain axis further complicates interpretation. Diet, microbiome composition, and cognitive function likely interact dynamically over time, thereby limiting the ability to establish directionality and disentangle causal pathways without longitudinal, microbiome-stratified, and temporally resolved study designs.

Taken together, the current body of evidence linking diet, the gut microbiota, and cognitive function is constrained by both methodological limitations and unresolved conceptual challenges. Although existing studies provide valuable insights, the field remains largely dependent on associative evidence and indirect mechanistic inference, with limited ability to establish causally coherent, human-relevant pathways.

Hence, future work should aim to integrate multi-omics approaches with longitudinal, microbiome-stratified study designs, including repeated sampling, standardized cognitive endpoints, and functionally relevant microbial measures. Without such advances, progress will remain limited to associative frameworks, restricting both mechanistic understanding and clinical translation.

## 4 Future directions and research perspectives

### 4.1 Multi-omics integration

Current approaches to studying diet–microbiome–brain interactions remain fundamentally constrained by an overreliance on taxonomic descriptions that inadequately capture functional activity. While multi-omics strategies, including metagenomics, metabolomics, transcriptomics, and proteomics, are frequently proposed as an effective solution, their implementation introduces substantial analytical and interpretative challenges, including issues related to data dimensionality, integration complexity, and reproducibility (Arıkan and Muth, 2023; Hasin et al., 2017; Knight et al., 2018).

Emerging microbiome-focused observational studies integrating microbiome sequencing with metabolomic profiling suggest that functional readouts, such as short-chain fatty acids and tryptophan-related metabolites, may provide more biologically informative links to brain-related outcomes than taxonomic composition alone. For example, population-based analyses have demonstrated associations between gut microbial composition, circulating microbial metabolites, and depression- and quality-of-life–related phenotypes, thereby providing indirect but relevant links to brain function (Valles-Colomer et al., 2019); however, studies directly integrating multi-omics data with domain-specific cognitive outcomes in humans remain limited, and findings are not yet consistent across populations or cognitive measures.

Moreover, the absence of standardized pipelines for multi-omics data integration, coupled with the continued reliance on cross-sectional sampling designs, further constrains causal inference and the ability to resolve temporally dynamic interactions. Although integrating multi-omics data with neuroimaging and detailed cognitive phenotyping represents a promising avenue toward improved functional resolution, its practical utility will depend on the development of harmonized analytical frameworks, such as machine learning–based integration models, network-based approaches, and mediation or multi-level modeling strategies, capable of robustly integrating complex biological datasets and improving causal inference between microbial activity and domain-specific cognitive outcomes (Tillisch et al., 2013).

For example, a longitudinal cohort design in which participants are followed over multiple time points, with repeated collection of dietary data, gut microbiome profiles, circulating metabolomic markers, and standardized cognitive assessments (e.g., Cognitive Abilities Screening Instrument [CASI]), alongside neuroimaging measures (e.g., functional MRI) where feasible. Analytical strategies would include dimensionality reduction techniques, cross-validated machine learning models, and appropriate correction for multiple testing (e.g., false discovery rate control) to minimize overfitting and enhance reproducibility in high-dimensional datasets (Hasin et al., 2017).

It is important to note that such approaches are unlikely to establish causal relationships in the absence of interventional designs but may substantially improve causal inference by identifying temporally coherent associations and biologically plausible pathways. Future research should therefore prioritize longitudinal, microbiome-stratified study designs incorporating repeated within-individual measurements, enabling the characterization of responder–nonresponder patterns and reducing confounding driven by inter-individual microbiome variability (Zmora et al., 2019). Furthermore, the selection of cognitive domains, including memory, executive function, and processing speed, in future studies, should be supported by evidence indicating their sensitivity to metabolic, inflammatory, and vascular pathways that may be influenced by gut microbial activity. Emerging microbiome-focused studies also suggest associations between gut microbial composition and brain-related outcomes; however, current evidence remains fragmented across cognitive and affective domains, with diet–cognition relationships and microbiome–brain associations often examined in separate contexts rather than within unified study designs (Saji et al., 2019; Valles-Colomer et al., 2019; Valls-Pedret et al., 2015).

For example, repeated profiling of microbial metabolites in conjunction with neuroimaging and harmonized cognitive testing may help clarify whether microbial dynamics track with specific alterations in memory, executive function, or processing speed over time, thereby advancing the field from associative observations toward more mechanistically informed, human-relevant interpretations.

### 4.2 Personalized nutrition

Microbiome-informed personalized nutrition has been widely proposed as a strategy to enhance efficacy; however, its translation into real-world clinical practice remains incompletely understood, particularly given challenges related to reproducibility, scalability, and host–microbiome heterogeneity (Kolodziejczyk et al., 2019; Vujkovic-Cvijin et al., 2020). Moreover, temporal instability in microbiome composition, together with the lack of standardized classification frameworks, further complicates the identification of stable and clinically actionable stratification markers (Kolodziejczyk et al., 2019; Zeevi et al., 2015).

Furthermore, baseline microbiome profiles are often assumed to predict individual responses to dietary interventions; however, accumulating evidence indicates that such predictions are inconsistent, context-dependent, and influenced by host and environmental factors (Vujkovic-Cvijin et al., 2020; Zeevi et al., 2015). While stratified randomized controlled trials may offer a pathway toward improved precision, their success will depend on robust, reproducible biomarkers that capture functionally relevant microbial activity, such as short-chain fatty acids, bile acids, and tryptophan-derived metabolites, rather than static compositional features (Nicholson et al., 2012).

### 4.3 Study design

The persistent reliance on cross-sectional and short-duration studies continues to constrain causal inference in this field. As such, these designs are inherently limited in capturing temporal dynamics and the directionality of diet–microbiome–brain interactions (Falony et al., 2016; Johnson et al., 2019). Although longitudinal cohort designs are frequently advocated, their implementation alone does not resolve key limitations, particularly in the absence of repeated microbiome and metabolomic measurements that capture within-individual variability over time (Johnson et al., 2019).

Similarly, randomized controlled trials remain limited by short intervention periods, insufficient statistical power, and inadequate consideration of baseline microbiome heterogeneity (Suez et al., 2018). These factors reduce the ability to detect sustained or clinically meaningful cognitive effects and may obscure responder-specific patterns, as has been observed in several nutrition and microbiome intervention studies reporting heterogeneous or domain-specific outcomes (Suez et al., 2018; Valles-Colomer et al., 2019). Advancing the field will require the development of study designs that integrate temporal resolution, microbiome stratification, and standardized domain-specific cognitive endpoints (e.g., memory, executive function, attention) within adequately powered frameworks.

### 4.4 Bridging causality gaps

A central challenge lies in distinguishing association from causation within complex, bidirectional gut–brain interactions. While analytical approaches such as mediation analysis and Mendelian randomization offer potential tools for causal inference, their application in this field remains constrained by strong and often untestable underlying assumptions (Davies et al., 2018; Sanna et al., 2019). In particular, Mendelian randomization relies on the absence of horizontal pleiotropy and the availability of valid genetic instruments; conditions that remain only partially satisfied in current microbiome genome-wide association studies (Kurilshikov et al., 2021).

Furthermore, causal inference frameworks are often insufficiently integrated with biological mechanisms, limiting interpretability and translational relevance, particularly when statistical associations are not supported by functional or mechanistic evidence (Nicholson et al., 2012). The incorporation of neuroimaging endpoints (e.g., functional MRI) and fluid biomarkers, such as neurofilament light chain and glial fibrillary acidic protein, may improve mechanistic resolution. However, the extent to which these markers capture microbiome-mediated effects on cognition remains incompletely characterized.

A practical framework for future research should combine repeated multi-omics sampling, microbiome-based participant stratification, standardized domain-specific cognitive endpoints, and longitudinal follow-up within a unified study design. Within this framework, future dietary intervention studies may also benefit from prospective microbiome-based participant stratification to identify individuals most likely to benefit from microbiota-targeted nutritional interventions and to better account for inter-individual variability in treatment response. Future work must move beyond descriptive and reductionist approaches toward analytically integrated, longitudinal, and functionally informed strategies that explicitly link microbial activity with cognitive outcomes. Without such advances, the field is likely to remain constrained to associative paradigms, with limited mechanistic resolution and translational relevance (Hasin et al., 2017; Tierney et al., 2019). An additional area requiring further investigation concerns sex-specific influences on microbiota–gut–brain interactions. Emerging evidence suggests that sex hormones, microbial composition, and immune responses may each differentially shape gut–brain communication in males and females (Caldarelli et al., 2024). However, current evidence in this area remains limited, and systematic sex-stratified investigation is notably absent from studies of neuroinflammation and cognitive aging, representing an important priority for future research (Caldarelli et al., 2024; Kuo et al., 2025).

## 5 Conclusion

The gut microbiota represents a central mediator linking dietary exposures to brain function through interconnected metabolic, immune, neural, and barrier-related pathways. Microbiota-derived metabolites, including short-chain fatty acids, tryptophan-derived compounds, and bile acids, play key roles in regulating inflammation, neurotransmission, and synaptic plasticity.

Diet-induced alterations in microbial composition are increasingly recognized to influence systemic inflammation, neuroinflammation, and cognitive decline. Conversely, dietary patterns that promote microbial diversity and beneficial metabolic outputs are consistently associated with more favorable cognitive trajectories.

While current evidence supports the biological plausibility of microbiota-mediated effects on brain health, translating these findings into clinically actionable strategies requires well-designed longitudinal studies, functionally informed multi-omics integration, and standardized cognitive assessment frameworks.

Emerging evidence indicates that aging remodels multiple components of the microbiota–gut–brain axis. Age-associated changes in microbial composition, reduced microbial metabolic capacity, inflammaging, impaired gut barrier integrity, and altered gut–brain communication may collectively increase vulnerability to neurodegenerative processes, highlighting the importance of considering aging mechanisms in future microbiome–brain research.

Advancing the field will depend on moving beyond associative evidence toward mechanistically integrated and temporally resolved models that capture the dynamic interplay between diet, microbial activity, immune regulation, and brain function. Such progress may ultimately enable the development of targeted, microbiome-informed nutritional strategies for promoting healthy cognitive aging, while maintaining appropriate caution in causal interpretation.
